# An Integrated Algorithm for Designing Oligodeoxynucleotides for Gene Synthesis

**DOI:** 10.3389/fgene.2022.836108

**Published:** 2022-03-17

**Authors:** Gang Fang, Hanjie Liang

**Affiliations:** Institute of Computing Science and Technology, Guangzhou University, Guangzhou, China

**Keywords:** gene synthesis, algorithm, oligodeoxynucleotide design, melting temperature, assembly

## Abstract

The design and construction of large synthetic genes can be a slow, difficult, and confusing process, especially in the key step of oligodeoxynucleotide design. Herein we present an integrated algorithm to design oligonucleotide sets for gene synthesis by both ligase chain reaction and polymerase chain reaction. It offers much flexibility with no constraints on the gene to be synthesized. Firstly, it divides the long-input DNA sequence by a greedy algorithm based on the length of the oligodeoxynucleotide overlap region. Secondly, it tunes the length of the overlap region iteratively in an attempt to minimize the melting temperature variance of overlap. Thirdly, dynamic programming algorithm is used to achieve the uniform melting temperature of the oligodeoxynucleotide overlaps. Finally, the oligodeoxynucleotides with homologous melting temperature necessary for ligase chain reaction-based or two-step assembly PCR-based synthesis of the desired gene are outputted.

## Introduction

Nowadays, gene synthesis is one of the most important biological technologies that can be used in the field of genome studies, gene expression studies, gene network studies, *etc*. This modern gene synthesis technique can synthesize a whole eukaryotic genome, and current gene synthesis methods rely on the use of overlapped oligonucleotides to construct large genes by ligase chain reaction (LCR) (Au et al., 1998) and polymerase chain reaction (PCR) (Stemmer et al., 1995). Algorithms and computer programs have been developed for gene synthesis to automatically design oligonucleotides to minimize the error of assembly and to optimize the LCR or PCR process. Programs such as TmPrime and DNAWorks, based on iteration algorithm, have been developed for gapless PCR assembly (David and Jacek, 2002; Marcus et al., 2009). Other programs, such as Gene2Oligo, Assembly PCR Oligo Maker, GeneDesign, and GeMS, also mainly based on iteration algorithm, have been developed for gap PCR assembly (Jean-Marie et al., 2004; Roman et al., 2005; Sebastian et al., 2005; Sarah et al., 2006). In the key step of oligodeoxynucleotide design, all the algorithms carried out in the programs will divide the input gene sequences into oligonucleotides with a homologous melting temperature, and the corresponding overlaps of these oligonucleotides possess uniform melting temperatures. The best result of these programs, which is attained by TmPrime, is less than 3°C in deviation of melting temperature (Marcus et al., 2009), but in optimization theory, it is not always the best solution to this sort of problem (Cormen et al., 2001). In order to prove this and minimize the error of assembly in gene synthesis, herein we present an integrated algorithm to solve this problem and attain a better result.

**FIGURE 1:**
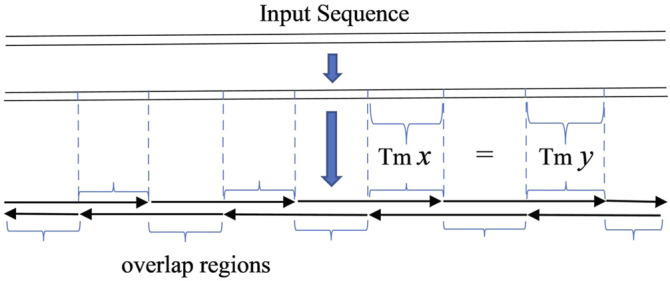
Scheme of gapless PCR or ligase chain reaction assembly. The input sequence is regarded as the serial connection of the overlap regions of oligonucleotides.

In the key step of oligodeoxynucleotide design in gene synthesis for gapless PCR or LCR assembly, all oligonucleotides are designed to be exactly adjacent, with no gap between two consecutive oligonucleotides. The given sequence can be seen as the serial connection of all overlapping regions of oligonucleotides. With this simple observation, the problem of designing oligonucleotides with a uniform melting temperature in overlaps will be equivalent to dividing the given sequence into segments with a homologous melting temperature, with each segment representing an overlapping region (Marcus et al., 2009) (Figure 1).

In gapped PCR assembly, oligonucleotides are adjacent, with few base deletions (gaps) between two consecutive oligonucleotides. Compared to gapless assembly, gapped assembly can be more flexible and economical, with an insignificant rise in assembly errors (Xiong et al., 2004), though gapped assembly can only be carried out by PCR. In this paper, the presented algorithm can output oligonucleotides with a homologous melting temperature, not only for gapless assembly but also for gapped assembly.

## Materials and Methods

Based on above-mentioned simple observation, the presented integrated algorithm firstly starts from a greedy algorithm. The greedy algorithm always selects the apparent best result in every step, without considering other options. Thus, it cannot always obtain the best solution to the problem although it is a fast algorithm (Cormen et al., 2001). Secondly, the result obtained from the initial greedy algorithm is optimized by iteration algorithm in an attempt to minimize the melting temperature variance of overlap regions. In this step, the best solution is also not guaranteed. In order to decrease the melting temperature variance of the overlap regions further, a dynamic programming algorithm is carried out. This dynamic algorithm, which is adapted from Viterbi algorithm, has been successfully fulfilled to solve other optimization problems in synthetic biology (Fang et al., 2017).

The initial greedy algorithm processes the input DNA sequence by dividing the sequence into segments with a similar melting temperature. It cuts down the first segment whose length is from 20 to 30 bp and then cuts down the second consecutive segment whose length is also from 20 to 30 bp. The melting temperature of these segments was computed, and the segment combination with the least deviation in melting temperature was selected. In this way, the first two segments are determined. In Table 1 the greedy algorithm is depicted in detail. Melting temperature is computed by using the nearest-neighbor model with SantaLucia’s thermodynamic parameter (SantaLucia and Hicks, 2004), corrected with salt and oligonucleotide concentrations, and the total number of phosphates in the duplex (Owczarzy et al., 2008).

**TABLE 1:** The Greedy algorithm.

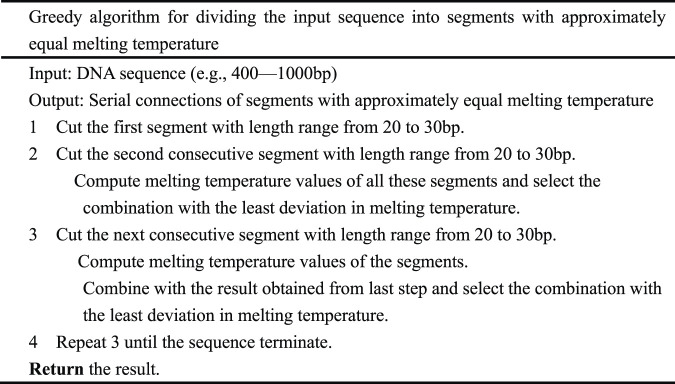

The greedy algorithm divides the input sequence into serial connections of segments with an approximately equal melting temperature. In theory, the algorithm cannot guarantee the best result. In an attempt to reduce the melting temperature variance of these serial connections of segments, iteration algorithm is carried out (Table 2).

**TABLE 2:** The iteration algorithm.

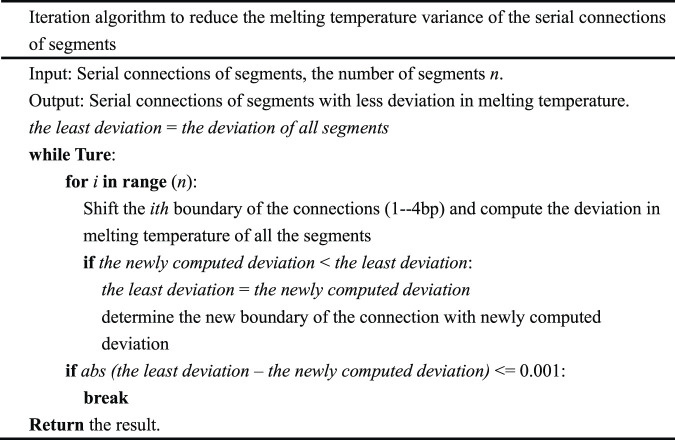

After the iteration algorithm, a better result will be given. The result can be used to produce oligodeoxynucleotides for LCR or gapless PCR assembly. In previous study, the PKB2 gene is selected for synthesis based on the reported difficulty of assembly *via* PCR (Gao et al., 2003). Compared to the oligodeoxynucleotide set that TmPrime produces for *Escherichia coli* codon-optimized PKB2 gene, the integrated algorithm presented in this paper can produce oligodeoxynucleotide sets for gapless PCR assembly with more uniform melting temperatures. The deviation in the melting temperature of oligodeoxynucleotide overlaps produced in this step by our algorithm is 0.6860 compared to 1.2004 in that produced by TmPrime (Supplementary Data S1).

In order to reduce the deviation in melting temperature of the overlap region further and produce more uniform oligodeoxynucleotide sets, a dynamic programming algorithm is employed. This type of algorithm is universally used in bioscience and other fields (Mount, 2001; Viterbi, 2006). In Table 3, the dynamic programming algorithm used to minimize the deviation in the melting temperature of oligodeoxynucleotide overlap regions is depicted in detail.

**TABLE 3:** The dynamic programming algorithm.

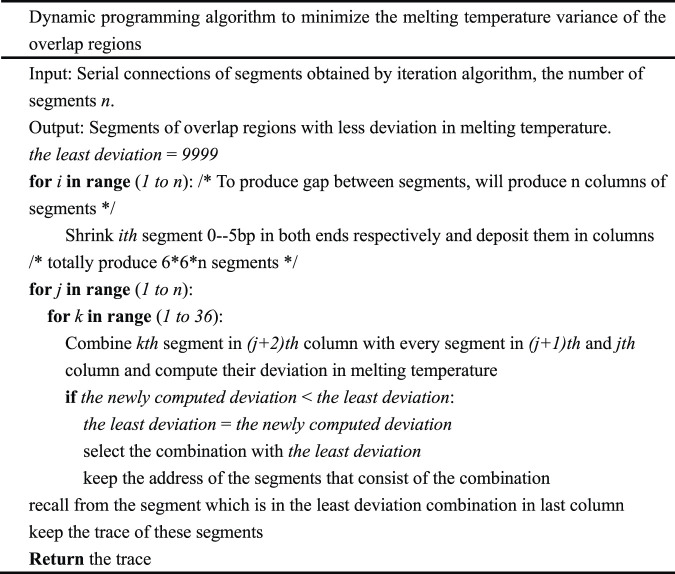

**FIGURE 2:**
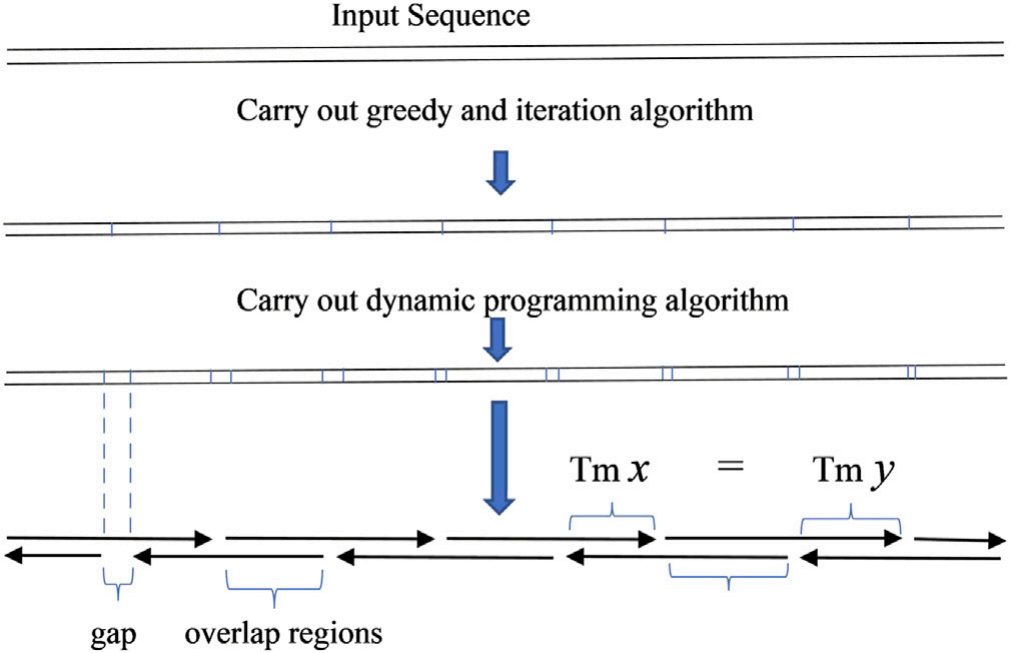
Scheme of gapped PCR assembly.

The result obtained from this algorithm can be used to produce oligodeoxynucleotides for gapped PCR assembly (Figure 2). Compared to the oligodeoxynucleotide sets that TmPrime produces for *E. coli* codon-optimized PKB2 gene, the result obtained by the dynamic programming algorithm can produce oligodeoxynucleotide sets for gapped PCR assembly with more uniform melting temperatures. The deviation in the melting temperature of oligodeoxynucleotide overlaps produced by the dynamic programming algorithm is 0.4944 compared to 1.2004 in that produced by TmPrime and 0.6860 by iteration algorithm (Supplementary Data S1). The presented integrated algorithm can produce oligodeoxynucleotide sets not only for gapless assembly but also for gapped assembly. If gapless assembly is needed, the result outputted by iteration algorithm can be used. If gapped assembly is needed, the step of iteration algorithm can be omitted, and the greedy algorithm and dynamic programming algorithm can just be integrated. In this way, the complexity of the whole algorithm can be decreased.

In order to produce an odd number of serial connections of segments, a small tail is added to the input sequence in some circumstances. This guarantees the imbricated structure of oligodeoxynucleotides as shown in diagrams in Figures 1, 2. The added tail can be eliminated in a PCR reaction by using particular primers.

## Results

When designing oligodeoxynucleotides for gene synthesis, the uniformity of oligo melting temperature especially in an overlap region is the key factor that should be considered. The oligodeoxynucleotide sets designed by the integrated algorithm possess the least SD in overlap melting temperature and can be used for gapless and gapped assembly (Table 4). The SD in melting temperature of the designed oligodeoxynucleotides is also less than what another program produces (Table 4). The deviation in melting temperature of oligodeoxynucleotides for the gapless assembly of PKB2 produced by iteration algorithm is 1.5102 compared to 1.7346 in that produced by TmPrime, and the deviation in melting temperature of oligodeoxynucleotides for gapped assembly is 1.4888. Dynamic programming will shrink each end of every fragment to produce candidate fragment columns. In fact, the final oligodeoxynucleotides for gapped assembly are adjacent, with a few base deletions (gaps) or with no gap between two consecutive oligonucleotides. This result is produced by the inherent property of dynamic programming algorithm. One can adjust this result by changing the number of bases to be shrunk in the first *for* loop of dynamic programming algorithm (refer to Supplementary Data S1 for more information). The algorithm is written in Python 3.7. The process of designing oligodeoxynucleotide sets for a multi-kilobase (<3 kb) gene takes less than 10 s when it is run on a Lenovo computer with dual 3.3-GHz Intel Xeons and 4 GB of RAM.

**TABLE 4:** Compared to the three genes designed by TmPrime, the oligonucleotide set designed by our algorithm possesses the least SD of overlap and oligonucleotides in Tm value (all the oligonucleotides were designed under the same conditions).

Gene	S100A4 (752 bp)		PKB2 (1,446 bp)		GFPuv (760 bp)	
	Overlap	Oligos	Overlap	Oligos	Overlap	Oligos
TmPrime	1.0980	1.5680	1.2004	1.7346	0.8660	0.9887
Ours (gapped)	0.2660	0.9690	0.4944	1.4888	0.1686	0.2796
Ours (gapless)	0.3890	1.0230	0.6860	1.5102	0.1890	0.3677

## Discussion

The integrated algorithm presented in this paper is fast and flexible, with no constraints on the input gene. It can design oligodeoxynucleotides for gene synthesis according to its inherent property. The annealing temperature of the assembly can be determined by the average melting temperature of the final oligodeoxynucleotide sets. The algorithm complexity of greedy algorithm is *O*(*n* ^*2*^) if the first two segments contain *n* options, respectively. The complexity of iteration algorithm is difficult to determine, but one can change the threshold of the difference between *the least deviation* and *the newly computed deviation* for a rapid convergence. According to our experience, the threshold that is set to 0.001 will make a rapid convergence. If the length of a serial connection of segments is *L* and the number of potential segments in a column is *N*, the algorithm complexity of dynamic programming algorithm will be *O*(*LN* ^*3*^). On the contrary, the algorithm complexity of exhaustive algorithm is *O*(*N* ^*L*^). The time complexity of dynamic programming algorithm can be more than *O*(*LN* ^*3*^), but for the reason of feasibility, we just consider the combination of three columns in the algorithm, which caused the time complexity to be *O*(*LN* ^*3*^) (Table 3). It is quite clear that the algorithm is technically feasible and can be run on an ordinary computing platform. Based on the simple observation, a greedy algorithm and iteration algorithm can be fulfilled to produce the oligodeoxynucleotides for gapless assembly. Despite the fact that a better result is obtained, in theory, the best solution to this sort of optimization problem cannot be guaranteed, and the best solution is extremely difficult to obtain. When dynamic programming algorithm is carried out after greedy and iteration algorithm, a better result than before is obtained. It can produce oligodeoxynucleotides with a more uniform melting temperature, and this will decrease the error of assembly, although it is only used for gapped PCR assembly. Compared to gapless assembly, gapped assembly may cause a rise in assembly error. The oligodeoxynucleotides designed by a dynamic programming algorithm possess a more uniform melting temperature, and this property can compensate the influence caused by the gaps between consecutive oligodeoxynucleotides. Furthermore, the number and the location of gaps can be adjusted by changing the number and the location of the bases to be shrunk in the first *for* loop of dynamic programming algorithm. The length of the oligodeoxynucleotides can be adjusted by changing the base number (length) of the segment to be cut in the beginning of the greedy algorithm (in this case, the base number to be cut is 20–30 bp, and it will produce an overlap length ranging from 20 to 30 bp, while the oligodeoxynucleotide length will approximately range from 40 to 60 bp. The dynamic programming algorithm is the core of this integrated algorithm. The first two algorithms just divide the input sequence approximately. The dynamic programming algorithm guarantees the uniformity of the melting temperature of the oligodeoxynucleotides that are produced for gene synthesis. Dynamic programming is universally used in optimization theory and solves a few optimization problems (Kececioglu and Myers, 1995). Its universality and feasibility lead to its application in the field of biology, especially in synthetic biology (Fang et al., 2017). This integrated algorithm will be simplified and written into a computer program, and a webserver will be built soon to facilitate the gene synthesis. Except the synchronization of the temperature of the overlap region, it is necessary to take into account other parameters, such as the presence of repeats, regions with high CG, *etc*. These factors will cause the formation of unwanted secondary structures. These problems have been successfully solved by FastPCR (Kalendar et al., 2011). In this paper, we emphasized the minimization of the melting temperature variance in the overlap region. We think that this can decrease the formation of unwanted secondary structures even if these cannot be eliminated. We do not consider other factors because we think that the minimization of the melting temperature variance in the overlap region can compensate for these to a certain extent.

## Data Availability

The original contributions presented in the study are included in the article/Supplementary Material, further inquiries can be directed to the corresponding author.
