# Supplementary material for: An Integrated Algorithm for Designing Oligodeoxynucleotides for Gene Synthesis
Source: Front Genet. 2022 Mar 17;13:836108. doi: 10.3389/fgene.2022.836108 (PMC8968678; doi:10.3389/fgene.2022.836108)
Supplement: Supplementary file 1 [file DataSheet1.PDF]

Supplementary Data

Oligonucleotides set designed for *E. coli* codon-optimized PKB2 (for gapped assembly).

lable	Oligonucleotide sequence (5' to 3')	len	Tm	overlap
F0	GAATGAGGTGTCTGTCATCAAAGAAGGCTGGCTCCACAAGCGTGGTGAAT	50	64.04	22
R0	CGTGGCCTCCAGGTCTTGATGTATTCACCACGCTTGTGGAGCCA	44	64.03	22
F1	ACATCAAGACCTGGAGGCCACGGTACTTCCTGCTGAAGAGCGACG	45	63.19	23
R1	CCTCTCCTTGTACCCAATGAAGGAGCCGTCGCTCTTCAGCAGGAAGTAC	49	65.2	26
F2	GCTCCTTCATTGGGTACAAGGAGAGGCCCGAGGCCCCTGATCAGACT	47	64.96	21
R2	ACGGAGAAGTTGTTTAAGGGGGGTAGAGTCTGATCAGGGGCCTCGGG	47	64.28	26
F3	CTACCCCCCTTAAACAACTTCTCCGTAGCAGAATGCCAGCTGATGAAGACC	51	65.27	25
R3	GTGTTGGGTCGCGGCCTCTCGGTCTTCATCAGCTGGCATTCTGCT	45	65.35	20
F4	GAGAGGCCGCGACCCAACACCTTTGTCATACGCTGCCTGCAGTG	44	64.66	24
R4	GAAGGTCCTCTCGATGACTGTGGTCCACTGCAGGCAGCGTATGACAAAG	49	64.64	25
F5	GACCACAGTCATCGAGAGGACCTTCCACGTGGATTCTCCAGACGAGAGG	49	64.67	24
R5	GATGGCCCGCATCCACTCCTCCCTCTCGTCTGGAGAATCCACGTG	45	64.95	21
F6	GAGGAGTGGATGCGGGCCATCCAGATGGTCGCCAACAGCCTCAA	44	65.0	23
R6	GCCTGGGGCCCGCTGCTTGAGGCTGTTGGCGACCATCTG	39	65.21	16
F7	GCAGCGGGCCCCAGGCGAGGACCCCATGGACTACAAGTGTG	41	64.91	25
R7	GGAGGAGTCACTGGGGGAGCCACACTTGTAGTCCATGGGGTCCTC	45	63.42	20
F8	GCTCCCCCAGTGACTCCTCCACGACTGAGGAGATGGAAGTGGC	43	63.78	23
R8	AGCCCGTGCCTTGCTGACCGCCACTTCCATCTCCTCAGTCGT	42	64.34	19
F9	GGTCAGCAAGGCACGGGCTAAAGTGACCATGAATGACTTCGACTATCTCAA	51	64.83	32
R9	CCAAAGGTTCCCTTGCCAAGGAGTTTGAGATAGTCGAAGTCATTCATGGTCACTTT	56	65.02	24
F10	ACTCCTTGGCAAGGGAACCTTTGGCAAAGTCATCCTGGTGCGGGAGAA	48	64.75	24
R10	CGTAGTAGCGGCCAGTGGCCTTCTCCCGCACCAGGATGACTTTG	44	64.87	20
F11	GGCCACTGGCCGCTACTACGCCATGAAGATCCTGCGAAAGGAAGTCA	47	65.16	27
R11	GTGAGCGACTTCATCCTTGGCAATGATGACTTCCTTTCGCAGGATCTTCATGG	53	64.71	25
F12	CATTGCCAAGGATGAAGTCGCTCACACAGTCACCGAGAGCCGGGTC	46	65.57	21
R12	CGGGTGCCTGGTGTTCTGGAGGACCCGGCTCTCGGTGACTGT	42	65.02	21
F13	CTCCAGAACACCAGGCACCCGTTCCTCACTGCGCTGAAGTATGCC	45	64.99	24
R13	AGGCGGTCGTGGGTCTGGAAGGCATACTTCAGCGCAGTGAGGAA	44	64.6	20
F14	TTCCAGACCCACGACCGCCTGTGCTTTGTGATGGAGTATGCCAACG	46	65.09	26
R14	CAGGTGGAAGAACAGCTCACCCCCGTTGGCATACTCCATCACAAAGCAC	49	65.21	23
F15	GGGGTGAGCTGTTCTTCCACCTGTCCCGGGAGCGTGTCTTCACA	44	64.79	21
R15	ATAAAACCGGGCCCGCTCCTCTGTGAAGACACGCTCCCGGGA	42	64.18	21
F16	GAGGAGCGGGCCCGGTTTTATGGTGCAGAGATTGTCTCGGCTCTT	45	64.51	24
R16	CGTCCCGCGAGTGCAAGTACTCAAGAGCCGAGACAATCTCTGCACC	46	65.42	22
F17	GAGTACTTGCACTCGCGGGACGTGGTATACCGCGACATCAAGCTGG	46	64.58	24

R17	GCCATCTTTGTCCAGCATGAGGTTTTCCAGCTTGATGTCGCGGTATACCA	50	64.32	26
F18	AAAACCTCATGCTGGACAAAGATGGCCACATCAAGATCACTGACTTTGGCCTCT	54	65.29	28
R18	CCCGTCACTGATGCCCTCTTTGCAGAGGCCAAAGTCAGTGATCTTGATGTG	51	65.99	23
F19	GCAAAGAGGGCATCAGTGACGGGGCCACCATGAAAACCTTCTGTGGG	47	64.48	24
R19	GCGCCAGGTACTCCGGGGTCCCACAGAAGGTTTTCATGGTGGC	43	65.57	19
F20	ACCCCGGAGTACCTGGCGCCTGAGGTGCTGGAGGACAATGACTATG	46	65.34	27
R20	CCAGTCCACGGCCCGGCCATAGTCATTGTCCTCCAGCACCTCAG	44	64.69	17
F21	GCCGGGCCGTGGACTGGTGGGGGCTGGGTGTGGTCAT	37	64.71	20
R21	GCGGCCGCACATCATCTCGTACATGACCACACCCAGCCCCCA	42	64.94	20
F22	ACGAGATGATGTGCGGCCGCCTGCCCTTCTACAACCAGGACCAC	44	65.17	24
R22	AGGATGAGCTCGAAGAGGCGCTCGTGGTCCTGGTTGTAGAAGGGCAG	47	64.93	22
F23	AGCGCCTCTTCGAGCTCATCCTCATGGAAGAGATCCGCTTCCCGCG	46	65.15	21
R23	GCCTCGGGGCTGAGCGTGCGCGGGAAGCGGATCTCTTCC	39	64.96	18
F24	CACGCTCAGCCCCGAGGCCAAGTCCCTGCTTGCTGGGCT	39	64.6	21
R24	GCCTCTGCTTGGGGTCCTTCTTAAGCAGCCCAGCAAGCAGGGACTTG	47	64.33	24
F25	TTAAGAAGGACCCCAAGCAGAGGCTTGGTGGGGGGCCCAGCG	42	64.1	16
R25	GTGCTCCATGACCTCCTTGGCATCGCTGGGCCCCCCACC	39	64.63	23
F26	ATGCCAAGGAGGTCATGGAGCACAGGTTCTTCCTCAGCATCAACTGGCA	49	65.18	25
R26	GGAGCTTCTTCTGGACCACGTCCTGCCAGTTGATGCTGAGGAAGAACC	48	64.94	23
F27	GGACGTGGTCCAGAAGAAGCTCCTGCCACCCTTCAAACCTCAGGTC	46	64.48	23
R27	GTACCTTGTGTCGACCTCGGACGTGACCTGAGGTTTGAAGGGTGGCA	47	64.83	23
F28	CGTCCGAGGTCGACACAAGGTACTTCGATGATGAATTTACCGCCCAGTCC	50	65.04	26
R28	CGGTCAGGGGGTGTGATTGTGATGGACTGGGCGGTAAATTCATCATCGA	49	64.31	23
F29	ATCACAATCACACCCCCTGACCGCTATGACAGCCTGGGCTTACTGGAG	48	64.78	25
R29	GAAGTGGGTCCGCTGGTCCAGCTCCAGTAAGCCCAGGCTGTCATAG	46	64.76	21
F30	CTGGACCAGCGGACCCACTTCCCCCAGTTCTCCTACTCGGCCA	43	65.11	22
R30	TGGCCGAGTAGGAGAACTGGGG	22	nan	nan
F_Primer	GAATGAGGTGTCTGTCATCAAAGAAGGC	28	nan	nan
R_Primer	TGGCCGAGTAGGAGAACTGGGG	28	nan	nan

```
overlap_tm: min:63.19, max:65.99, Range:2.8, mean:64.8, std:0.4944
gene_tm: min:78.41, max:85.32, Range:6.9, mean:81.8, std:1.4888
```

Oligonucleotides set designed for *E. coli* codon-optimized PKB2 (for gapless assembly).

lable	Oligonucleotide sequence (5' to 3')	len	Tm	overlap
F0	GAATGAGGTGTCTGTCATCAAAGAAGGCTGGCTCCACAAGCGTGGTGAAT	50	64.04	22
R0	CGTGGCCTCCAGGTCTTGATGTATTCACCACGCTTGTGGAGCCA	44	64.03	22
F1	ACATCAAGACCTGGAGGCCACGGTACTTCCTGCTGAAGAGCGACG	45	63.19	23
R1	CCTCTCCTTGTACCCAATGAAGGAGCCGTCGCTCTTCAGCAGGAAGTAC	49	65.2	26

F2	GCTCCTTCATTGGGTACAAGGAGAGGCCCGAGGCCCCTGATCAGACT	47	64.96	21
R2	ACGGAGAAGTTGTTTAAGGGGGGTAGAGTCTGATCAGGGGCCTCGGG	47	64.28	26
F3	CTACCCCCCTTAAACAACTTCTCCGTAGCAGAATGCCAGCTGATGAAGACC	51	65.27	25
R3	GTGTTGGGTCGCGGCCTCTCGGTCTTCATCAGCTGGCATTCTGCT	45	65.35	20
F4	GAGAGGCCGCGACCCAACACCTTTGTCATACGCTGCCTGCAGTG	44	64.66	24
R4	GAAGGTCCTCTCGATGACTGTGGTCCACTGCAGGCAGCGTATGACAAAG	49	64.64	25
F5	GACCACAGTCATCGAGAGGACCTTCCACGTGGATTCTCCAGACGAGAGG	49	64.67	24
R5	GATGGCCCGCATCCACTCCTCCCTCTCGTCTGGAGAATCCACGTG	45	64.95	21
F6	GAGGAGTGGATGCGGGCCATCCAGATGGTCGCCAACAGCCTCAA	44	65.0	23
R6	GCCTGGGGCCCGCTGCTTGAGGCTGTTGGCGACCATCTG	39	65.21	16
F7	GCAGCGGGCCCCAGGCGAGGACCCCATGGACTACAAGTGTG	41	64.91	25
R7	GGAGGAGTCACTGGGGGAGCCACACTTGTAGTCCATGGGGTCCTC	45	63.42	20
F8	GCTCCCCCAGTGACTCCTCCACGACTGAGGAGATGGAAGTGGC	43	63.78	23
R8	AGCCCGTGCCTTGCTGACCGCCACTTCCATCTCCTCAGTCGT	42	64.34	19
F9	GGTCAGCAAGGCACGGGCTAAAGTGACCATGAATGACTTCGACTATCTCAA	51	64.83	32
R9	CCAAAGGTTCCCTTGCCAAGGAGTTTGAGATAGTCGAAGTCATTCATGGTCACTTT	56	65.02	24
F10	ACTCCTTGGCAAGGGAACCTTTGGCAAAGTCATCCTGGTGCGGGAGAA	48	64.75	24
R10	CGTAGTAGCGGCCAGTGGCCTTCTCCCGCACCAGGATGACTTTG	44	64.87	20
F11	GGCCACTGGCCGCTACTACGCCATGAAGATCCTGCGAAAGGAAGTCAT	48	65.39	28
R11	GTGAGCGACTTCATCCTTGGCAATGATGACTTCCTTTCGCAGGATCTTCATGG	53	64.71	25
F12	CATTGCCAAGGATGAAGTCGCTCACACAGTCACCGAGAGCCGGGTC	46	65.57	21
R12	CGGGTGCCTGGTGTTCTGGAGGACCCGGCTCTCGGTGACTGT	42	65.02	21
F13	CTCCAGAACACCAGGCACCCGTTCCTCACTGCGCTGAAGTATGCC	45	64.99	24
R13	AGGCGGTCGTGGGTCTGGAAGGCATACTTCAGCGCAGTGAGGAA	44	64.6	20
F14	TTCCAGACCCACGACCGCCTGTGCTTTGTGATGGAGTATGCCAACG	46	65.09	26
R14	CAGGTGGAAGAACAGCTCACCCCCGTTGGCATACTCCATCACAAAGCAC	49	65.21	23
F15	GGGGTGAGCTGTTCTTCCACCTGTCCCGGGAGCGTGTCTTCACA	44	64.79	21
R15	ATAAAACCGGGCCCGCTCCTCTGTGAAGACACGCTCCCGGGA	42	64.18	21
F16	GAGGAGCGGGCCCGGTTTTATGGTGCAGAGATTGTCTCGGCTCTT	45	64.51	24
R16	CGTCCCGCGAGTGCAAGTACTCAAGAGCCGAGACAATCTCTGCACC	46	65.42	22
F17	GAGTACTTGCACTCGCGGGACGTGGTATACCGCGACATCAAGCTGG	46	64.58	24
R17	GCCATCTTTGTCCAGCATGAGGTTTTCCAGCTTGATGTCGCGGTATACCA	50	64.32	26
F18	AAAACCTCATGCTGGACAAAGATGGCCACATCAAGATCACTGACTTTGGCCTCT	54	65.29	28
R18	CCCGTCACTGATGCCCTCTTTGCAGAGGCCAAAGTCAGTGATCTTGATGTG	51	65.99	23
F19	GCAAAGAGGGCATCAGTGACGGGGCCACCATGAAAACCTTCTGTGGG	47	64.48	24
R19	GCGCCAGGTACTCCGGGGTCCCACAGAAGGTTTTCATGGTGGC	43	65.57	19
F20	ACCCCGGAGTACCTGGCGCCTGAGGTGCTGGAGGACAATGACTATG	46	65.34	27
R20	CCAGTCCACGGCCCGGCCATAGTCATTGTCCTCCAGCACCTCAG	44	64.69	17
F21	GCCGGGCCGTGGACTGGTGGGGGCTGGGTGTGGTCAT	37	64.71	20
R21	GCGGCCGCACATCATCTCGTACATGACCACACCCAGCCCCCA	42	66.06	22
F22	GTACGAGATGATGTGCGGCCGCCTGCCCTTCTACAACCAGGACCAC	46	65.17	24
R22	GAGGATGAGCTCGAAGAGGCGCTCGTGGTCCTGGTTGTAGAAGGGCAG	48	67.11	24
F23	GAGCGCCTCTTCGAGCTCATCCTCATGGAAGAGATCCGCTTCCCGCG	47	66.62	23

R23	GCCTCGGGGCTGAGCGTGCGCGGGAAGCGGATCTCTTCCAT	41	64.96	18
F24	CACGCTCAGCCCCGAGGCCAAGTCCCTGCTTGCTGGGCTG	40	66.07	22
R24	GCCTCTGCTTGGGGTCCTTCTTAAGCAGCCCAGCAAGCAGGGACTTG	47	65.46	25
F25	CTTAAGAAGGACCCCAAGCAGAGGCTTGGTGGGGGGCCCAGCG	43	66.34	18
R25	TGTGCTCCATGACCTCCTTGGCATCGCTGGGCCCCCCACCAA	42	65.82	24
F26	ATGCCAAGGAGGTCATGGAGCACAGGTTCTTCCTCAGCATCAACTGGCA	49	65.18	25
R26	GGAGCTTCTTCTGGACCACGTCCTGCCAGTTGATGCTGAGGAAGAACC	48	64.94	23
F27	GGACGTGGTCCAGAAGAAGCTCCTGCCACCCTTCAAACCTCAGGTCA	47	65.69	24
R27	GTACCTTGTGTCGACCTCGGACGTGACCTGAGGTTTGAAGGGTGGCA	47	64.83	23
F28	CGTCCGAGGTCGACACAAGGTACTTCGATGATGAATTTACCGCCCAGTCC	50	65.43	27
R28	CGGTCAGGGGGTGTGATTGTGATGGACTGGGCGGTAAATTCATCATCGAA	50	64.31	23
F29	ATCACAATCACACCCCCTGACCGCTATGACAGCCTGGGCTTACTGGAG	48	64.78	25
R29	GAAGTGGGTCCGCTGGTCCAGCTCCAGTAAGCCCAGGCTGTCATAG	46	64.76	21
F30	CTGGACCAGCGGACCCACTTCCCCCAGTTCTCCTACTCGGCCA	43	65.11	22
R30	TGGCCGAGTAGGAGAACTGGGG	22	nan	nan
F_Primer	GAATGAGGTGTCTGTCATCAAAGAAGGC	28	nan	nan
R_Primer	TGGCCGAGTAGGAGAACTGGGG	28	nan	nan

```
overlap_tm: min:63.19, max:67.11, Range:3.9, mean:65.0, std:0.6860
gene_tm: min:78.41, max:85.32, Range:6.9, mean:81.8, std:1.5102
```

The oligonucleotides set designed by TmPrime for *E. coli* codon-optimized PKB2 (for gapless assembly).

Label	Oligonucleotide sequence (5' to 3')	T_m (°C)	Overlap (bp)	Length (nt)
F0	ATGAATGAGGTGTCTGTCATCAAAGAAGGCTGGCTCCACAAGCGTGGTG	64.3	20	49
R0	CGTGGCCTCCAGGTCTTGATGTATTCACCACGCTTGTGGAGCCAG	64.9	25	45
F1	AATACATCAAGACCTGGAGGCCACGGTACTTCCTGCTGAAGAGCGACG	63.6	23	48
R1	CCTCTCCTTGTACCCAATGAAGGAGCCGTCGCTCTTCAGCAGGAAGTAC	65.0	26	49
F2	GCTCCTTCATTGGGTACAAGGAGAGGCCCGAGGCCCCTGATCAGAC	64.5	20	46
R2	ACGGAGAAGTTGTTTAAGGGGGGTAGAGTCTGATCAGGGGCCTCGGG	65.4	27	47
F3	TCTACCCCCCTTAAACAACTTCTCCGTAGCAGAATGCCAGCTGATGAAGACC	65.6	25	52
R3	GTGTTGGGTCGCGGCCTCTCGGTCTTCATCAGCTGGCATTCTGCT	66.1	20	45
F4	GAGAGGCCGCGACCCAACACCTTTGTCATACGCTGCCTGCAGTGG	66.7	25	45
R4	GTGGAAGGTCCTCTCGATGACTGTGGTCCACTGCAGGCAGCGTATGACAAAG	67.7	27	52
F5	ACCACAGTCATCGAGAGGACCTTCCACGTGGATTCTCCAGACGAGAGGGAGGAG	67.4	27	54
R5	CCATCTGGATGGCCCGCATCCACTCCTCCCTCTCGTCTGGAGAATCCAC	67.3	22	49
F6	TGGATGCGGGCCATCCAGATGGTCGCCAACAGCCTCAAGCAGC	66.4	21	43
R6	GTCCTCGCCTGGGGCCCGCTGCTTGAGGCTGTTGGCGA	65.1	17	38

F7	GGGCCCCAGGCGAGGACCCCATGGACTACAAGTGTGGCTCCC	67.1	25	42
R7	TCCTCAGTCGTGGAGGAGTCACTGGGGGAGCCACACTTGTAGTCCATGGG	67.3	25	50
F8	CCAGTGACTCCTCCACGACTGAGGAGATGGAAGTGGCGGTCAGCAAGG	66.3	23	48
R8	GTCATTCATGGTCACTTTAGCCCGTGCCTTGCTGACCGCCACTTCCATC	64.4	26	49
F9	CACGGGCTAAAGTGACCATGAATGACTTCGACTATCTCAAACTCCTTGGCAAGG	65.0	28	54
R9	CACCAGGATGACTTTGCCAAAGGTTCCCTTGCCAAGGAGTTTGAGATAGTCGAA	64.8	26	54
F10	GAACCTTTGGCAAAGTCATCCTGGTGCGGGAGAAGGCCACTGGCC	65.6	19	45
R10	CGCAGGATCTTCATGGCGTAGTAGCGGCCAGTGGCCTTCTCCCG	65.7	25	44
F11	GCTACTACGCCATGAAGATCCTGCGAAAGGAAGTCATCATTGCCAAGGATGAAGTC	65.8	31	56
R11	GGCTCTCGGTGACTGTGTGAGCGACTTCATCCTTGGCAATGATGACTTCCTTT	65.6	22	53
F12	GCTCACACAGTCACCGAGAGCCGGGTCCTCCAGAACACCAGGCA	66.5	22	44
R12	CTTCAGCGCAGTGAGGAACGGGTGCCTGGTGTTCTGGAGGACCC	66.2	22	44
F13	CCCGTTCCTCACTGCGCTGAAGTATGCCTTCCAGACCCACGACCG	66.6	23	45
R13	GTTGGCATACTCCATCACAAAGCACAGGCGGTCGTGGGTCTGGAAGGCATA	66.6	28	51
F14	CCTGTGCTTTGTGATGGAGTATGCCAACGGGGGTGAGCTGTTCTTCCACCT	66.5	23	51
R14	TGTGAAGACACGCTCCCGGGACAGGTGGAAGAACAGCTCACCCCC	67.0	22	45
F15	GTCCCGGGAGCGTGTCTTCACAGAGGAGCGGGCCCGGTTTTATG	66.1	22	44
R15	TACTCAAGAGCCGAGACAATCTCTGCACCATAAAACCGGGCCCGCTCCTC	66.4	28	50
F16	GTGCAGAGATTGTCTCGGCTCTTGAGTACTTGCACTCGCGGGACGTGG	66.6	20	48
R16	GGTTTTCCAGCTTGATGTCGCGGTATACCACGTCCCGCGAGTGCAAG	65.7	27	47
F17	TATACCGCGACATCAAGCTGGAAAACCTCATGCTGGACAAAGATGGCCACATC	65.9	26	53
R17	TCTTTGCAGAGGCCAAAGTCAGTGATCTTGATGTGGCCATCTTTGTCCAGCATGA	66.8	29	55
F18	AAGATCACTGACTTTGGCCTCTGCAAAGAGGGCATCAGTGACGGGGCCA	67.5	20	49
R18	CGGGGTCCCACAGAAGGTTTTCATGGTGGCCCCGTCACTGATGCCC	67.8	26	46
F19	CCATGAAAACCTTCTGTGGGACCCCGGAGTACCTGGCGCCTGAGGTGC	67.6	22	48
R19	GCCCGGCCATAGTCATTGTCCTCCAGCACCTCAGGCGCCAGGTACTC	68.7	25	47
F20	TGGAGGACAATGACTATGGCCGGGCCGTGGACTGGTGGGGGCTGG	67.7	20	45
R20	CCGCACATCATCTCGTACATGACCACACCCAGCCCCCACCAGTCCACG	67.6	28	48
F21	GTGTGGTCATGTACGAGATGATGTGCGGCCGCCTGCCCTTCTACAACCAGGA	68.6	24	52
R21	GAGCTCGAAGAGGCGCTCGTGGTCCTGGTTGTAGAAGGGCAGGCGG	67.8	22	46
F22	CCACGAGCGCCTCTTCGAGCTCATCCTCATGGAAGAGATCCGCTTCCCG	67.9	27	49
R22	CCTCGGGGCTGAGCGTGCGCGGGAAGCGGATCTCTTCCATGAGGAT	68.6	19	46
F23	CGCACGCTCAGCCCCGAGGCCAAGTCCCTGCTTGCTGGGCT	67.7	22	41
R23	GCCTCTGCTTGGGGTCCTTCTTAAGCAGCCCAGCAAGCAGGGACTTGG	67.6	26	48
F24	GCTTAAGAAGGACCCCAAGCAGAGGCTTGGTGGGGGGCCCAGCG	67.9	18	44
R24	CTGTGCTCCATGACCTCCTTGGCATCGCTGGGCCCCCCACCAA	67.1	25	43
F25	ATGCCAAGGAGGTCATGGAGCACAGGTTCTTCCTCAGCATCAACTGGCAGGA	67.0	27	52
R25	GGCAGGAGCTTCTTCTGGACCACGTCCTGCCAGTTGATGCTGAGGAAGAAC	67.5	24	51
F26	CGTGGTCCAGAAGAAGCTCCTGCCACCCTTCAAACCTCAGGTCACGTCC	66.7	25	49

R26	TCATCATCGAAGTACCTTGTGTCGACCTCGGACGTGACCTGAGGTTTGAAGGGT	66.2	29	54
F27	GAGGTCGACACAAGGTACTTCGATGATGAATTTACCGCCCAGTCCATCACAATCACA	66.9	28	57
R27	AGGCTGTCATAGCGGTCAGGGGGTGTGATTGTGATGGACTGGGCGGTAAAT	68.3	23	51
F28	CCCCCTGACCGCTATGACAGCCTGGGCTTACTGGAGCTGGACCAGC	67.4	23	46
R28	AGTAGGAGAACTGGGGGAAGTGGGTCCGCTGGTCCAGCTCCAGTAAGCCC	68.8	27	50
F29	GGACCCACTTCCCCCAGTTCTCCTACTCGGCCAGCATCCGCGAGTGA	68.0	20	47
R29	TCACTCGCGGATGCTGGCCG			20
F_Primer	ATGAATGAGGTGTCTGTCATCAAAGAAGG	63.6		29
R_Primer	TCACTCGCGGATGCTGGCCG	68.0		20

Its overlap region and oligonucleotides Tm value statistics:

```
old_tm: min:63.6, max:68.8, Range:5.2, mean:66.6, std:1.2004
overlap_tm: min:63.19, max:69.11, Range:5.9, mean:66.3, std:1.3120
gene_tm: min:78.89, max:86.45, Range:7.6, mean:82.5, std:1.7346
```

The first line is the overlap statistics and the third line is the oligonucleotides statistics.
